# Infantile neurodevelopmental outcome after fetoscopic laser photocoagulation for twin-to-twin transfusion syndrome: the first prospective experience from Iran

**DOI:** 10.1186/s12884-022-04793-x

**Published:** 2022-06-01

**Authors:** Nazila Mesbah, Vajiheh Marsousi, Laleh Eslamian, Hadi Montazerlotfelahi, Alireza A. Shamshirsaz, Kamran Hessami, Ashraf Jamal, Maryam Noorzadeh, Mahsa Naemi, Marjan Ghaemi

**Affiliations:** 1 Department of Obstetrics and Gynecology, Shariati Hospital, Tehran University of Medical Sciences, Tehran, Iran; 2 Department of Pediatrics, Imam Ali Hospital, Alborz University of Medical Sciences, Karaj, Iran; 3 Maternal Fetal Care Center, Boston Children’s Hospital, Harvard Medical School, Boston, MA USA; 4 Vali-E-Asr Reproductive Health Research Center, Family Health Research Institute, Tehran University of Medical Sciences, Tehran, Iran

**Keywords:** Twin pregnancy, Monochorionic, Twin twin transfusion syndrome, Neurodevelopment, Fetoscopic laser photocoagulation

## Abstract

**Objective:**

We sought to evaluate the neurodevelopmental outcomes at 12 months of age among infants with twin-to-twin transfusion syndrome (TTTS) undergoing fetoscopic laser photocoagulation (FLP).

**Materials and methods:**

In this prospective longitudinal study, neurodevelopmental assessment was performed among the infants at the corrected age of 12 months, who were diagnosed with TTTS and treated by FLP. The Ages and Stages Questionnaire (ASQ) was filled out by parents. In the next step in infants with abnormal ASQ, motor and cognitive developments were evaluated by Bayley’s infant and toddler development scoring system (Bayley 3-Third edition).

**Results:**

In 39 FLP procedures the rate of live birth of at least one twin was 73.8%. Four neonatal deaths were recorded, three of which were due to prematurity and one was due to heart anomaly. The ASQ was normal in 89.7% (35/39) of the infants (group I), 5.1% (2/39) had minor neurodevelopmental impairment (NDI) (group II), and 5.1% (2/39) had major NDI (group III). The 4 infants with abnormal ASQ had Bayley examination which showed two with mild to moderate cerebral palsy and two had delayed verbal skills and autistic spectrum disorder. No significant difference was noted between survivors with and without NDI with respect to donor or recipient status, birth weight, gestational age at birth, Quintero stage of TTTS. In addition, the relationship between gestational age at the time of undergoing FLC and NDI was not significant.

**Conclusion:**

In our population, minor and major neurodevelopmental impairment were seen in 10.2% of the infants. This information is useful for counseling our couples in this population prior the procedure.

## Introduction

The incidence of twin pregnancies has increased over the past three decades due to various factors such as advanced maternal age at the time of conception and the use of assisted reproductive technologies (ARTs) [1]. Twin-to-twin transfusion syndrome (TTTS) is a serious condition, which affects 10–15% of monochorionic multiple pregnancies.

Fetoscopic laser photocoagulation (FLP) of the placental vascular anastomoses has been introduced as standard therapeutic strategy. Fetoscopic laser coagulation ablates placental vascular anastomoses between twins and transforms the monochorionic twins into dichorionic ones [2] and since its introduction, it has been associated with significant improvement in perinatal survival [3]. A recent meta-analysis evaluating 1,499 TTTS survivors revealed that the overall incidence of neurodevelopmental impairment (NDI) is 14.0% among survivors and factors such as later gestational age at the time of FLP, earlier gestational age at delivery and lower birth weight may increase the risk of neurodevelopmental impairment [4]. Neurodevelopment involves a number of areas, any of which may be impaired either in isolation or in combination. In a review study which summarized the neurodevelopmental outcomes of the related studies performed during 17 years, the rate of cerebral palsy following laser treatment ranged between 3 and 12%, and the rate of neurodevelopmental impairment (i.e., cerebral palsy, severe cognitive and/or motor delay (< 2 SD), blindness, and/or deafness) spanned from 4 to 18% [5].

Survival of one and more fetuses following FLP is more than 90% and 70% respectively, however we do not have much information about the long-term potential effect of this treatment on the neurodevelopmental status of the newborns. Prior to laser therapy, at least one in five survivors of TTTS had serious adverse neurodevelopmental outcomes such as usually cerebral palsy. Current estimates of neurological impairment among survivors following laser surgery vary from 4 to 31% and long-term follow-up data are limited [6]. Therefore, this study is aimed to define the long-term neurologic consequences of this therapy in infants born of TTTS monochorionic twin pregnancies treated by FCL and its relative factors.

## Materials and methods

### Setting

This prospective longitudinal study was performed at Shariati Hospital affiliated to Tehran University of Medical Sciences, Tehran, Iran, from October 2018 to June 2021. The infants aged 12 months old who were born from monochorionic twin pregnancies diagnosed with TTTS and treated with FLP were enrolled in this study.

Since the establishment of the FLP center at Shariati Hospital in Tehran, all TTTS cases in Iran have been referred to this center, as it is the only active FLP center in the country. This study was in accordance with the ethical issues for human subject's research and confirmed by the Tehran University of Medical Sciences review of board. Informed consent was obtained from all subjects.

### Inclusion/exclusion

The inclusion criteria consisted of a child born of a TTTS monochorionic diamniotic twin pregnancy that all were treated with FLP and followed for neurodevelopmental assessment at a corrected age of at least 12 months after study. The exclusion criterion was children who may not returned for followed up in our center. The severity of TTTS II to IV was stage base on Quintero’s classification [7]. Also, we omitted the triplet pregnancies.

FLP was performed under general or spinal anesthesia with a small skin incision at the best insertion site followed by the percutaneous insertion of trocar into the amniotic cavity, and then a 3.3-mm fetoscope (11506AA Karl Storz, Germany) was inserted. Fetoscope insertion was performed by the Seldinger technique. In pregnancies with anterior placenta, we used a curved trocar of the same size and diameter. All the superficial intertwin placental anastomosis cases, including arteriovenous (AV) arterioarterial (AA), and venovenous (VV), were directly identified and coagulated with NdYag laser (400–600 micron laser fiber). At the end of FLC, after removing the fetoscope, the amniotic fluid was drained through the sheath until a vertical pool of 6 cm was confirmed.

After the procedure, the mothers were admitted to the prenatal ward for 24 h, and indomethacin (100 mg rectal suppository), nifedipine (10 mg oral), ceftriaxone (1 gr IV BD for 24 h), and Proluton Depot (250 mg IM) (injectable form progesterone) were administered. Twenty-four hours later, the fetuses were examined by ultrasonography, and uncomplicated mothers were discharged. They were requested to come back to the hospital after two weeks to recheck, and an ultrasound was performed for the early detection of twin anemia polycythemia sequence (TAPS), the recurrence of TTTS, and growth disorder. We did not detect any iatrogenic TAPS or TTTS in our patients. All the mothers were asked to visit the perinatologist every two weeks until delivery.

All the fetuses were delivered in the local referring hospital. Delivery was planned according to the obstetric indication, growth, and Doppler parameters. Besides, the pregnancy, fetal, and neonatal outcomes were evaluated as the secondary outcomes in this study. Also, autism spectrum disorder (ASD) was evaluated in the infants by Bayley scale. ASD is defined as a neurodevelopmental disorder by deficits in social communication and the presence of restricted interests and repetitive behaviors [8] that was categorized as group II in the current study.

### ASQ questionnaire

The infants were examined neurologically and physically by a pediatrician, and the ASQ (specific for 12 months) questionnaire was filled out by the parents. The ASQ was developed by J. Squires and D. Bricker and can be completed by parents in 12–18 min. The ASQ-3 is a parent-reported initial level developmental screening instrument consisting of 21 intervals, each with 30 items in five areas: (l) personal-social, (ll) gross motor, (lll) fine motor, [9] problem solving, and (V) communication for children aged 2–60 months. The infants were referred for further evaluation if 1. Children who score in at least one developmental area equal to or less than the cut-off points of -2 SD (two deviations below average). 2. At the first visit, the score was between 1SD and SD, and after doing so evolutionary measures at home, after 2 weeks, re-testing is still less than 1SD. 3. The pediatrician think the infant is not normal.

### Bayley examination

In the next step in infants with abnormal ASQ, motor and cognitive developments were evaluated by Bayley’s infant and toddler development scoring system (Bayley 3-Third edition). Neurodevelopmental impairment (ND) was defined as at least one of the following: CP, severe motor and/or cognitive developmental delay, bilateral blindness, or deafness requiring amplification with hearing aids [5]. The standard Bayley examination is used to evaluate the development rate in neonates. The 0–100 scoring system investigates five skills in neonates, including cognitive, language (Receptive & Expressive), motor (Gross & fine), social-emotional, and adaptive behaviors. If any developmental impairment was detected, they were categorized by Gross Motor Function Classification System (GMFCS). Based on standard scores, the BSID classifies performance into one of the following four categories to provide an indication of development: (a) accelerated development, (b) within normal limits, (c) mildly delayed, and (d) significantly delayed, with lower scores indicating greater impairment. These standard scores can be also converted to reflect age equivalence. Afterward, risk factors associated with mild and severe impairment were compared to determine which risk factors affected motor and cognitive numbers in Bayley 3.

### Statistical analysis

According to the neurologic examinations, all the data were classified into three groups as follow: Group I with normal neurological examination, group II with minor neurological abnormalities and group III with major neurological abnormalities including cerebral palsy [10].

To analyze the data, we used descriptive statistics, including means and percentages, and Pearson’s correlation coefficient, Chi-squared test, Fisher’s exact test (when *n* < 5), and two-factor analysis of variance (ANOVA). For quantitative variables, Student’s *t*-test or Mann–Whitney U test was used in compliance with their applicability conditions. After collecting the data, analysis was done with SPSS version 22. A *P*-value of less than 0.05 was considered significant.

## Results

In total, 40 (80 fetuses) monochorionic diamniotic pregnancies diagnosed with TTTS were recruited in this study. The procedure was successful in 100% of the procedures.

**Fig. 1:**
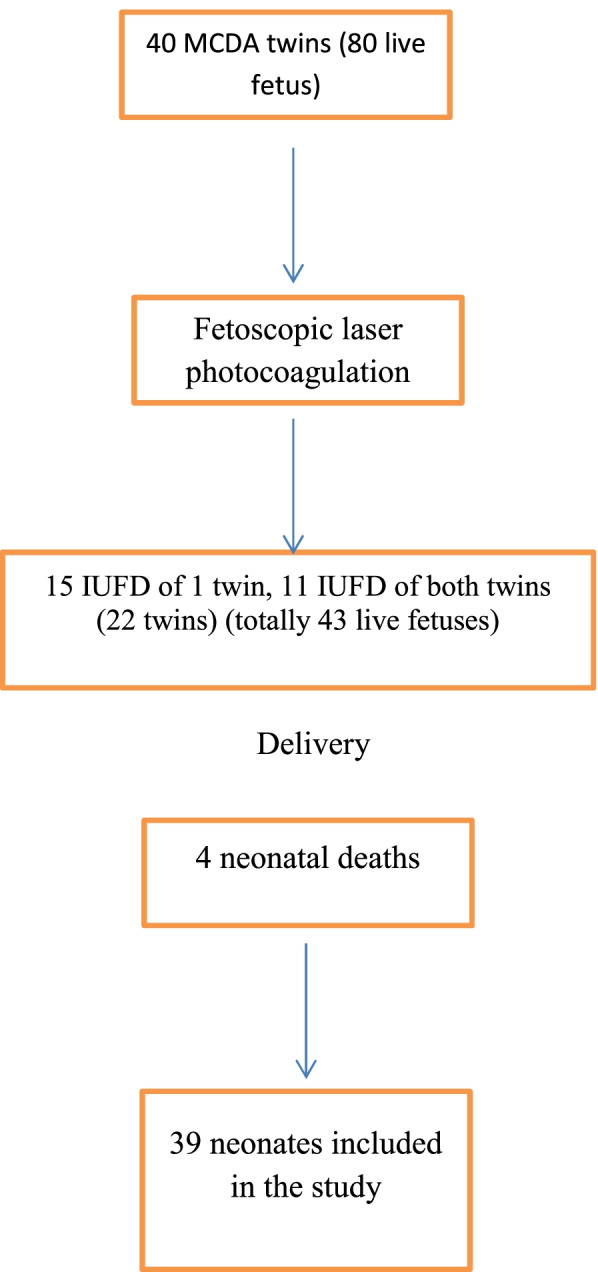
The diagram recruiting cases of study

As is shown in Fig. 1, 43 neonates consisted of 14 pairs of live twins (28 fetuses), 15 live of one twin (either donor or recipient) (15 fetuses) were considered for further evaluation. The live birth of at least one twin in all of the procedures was 73% of the pregnancies (29/40). Fetal and neonatal characteristics of the cases are summarized in Table 1. The information of infants with TTTS and age at birth are presented in Table 2.

**Table 1:** Maternal, fetal and neonatal characteristics of the participants (*n* = 43)

Characteristics	Mean ± SD
Maternal age (year)	29.4 (18_41y) ^a^
Gestational age at laser (week)	21.49 ± 2.24
Birth Weight (gram)	1767.1 ± 519.5

^a^range

**Table 2:** Infants’ gestational age at birth and the stage of TTTS information (*n* = 43)

Variables	Frequency (%)
**Gestational age at birth**	
26–27^+6^ week	3 (6.9)
28–29^+6^ week	8 (18.6)
30–31^+6^ week	14 (32.5)
32–36^+6^ week	15 (34.8)
37 < week	3 (6.9)
**Stage of TTTS**	
II	26 (60.4)
III	15 (34.8)
IV	2 (4.6)

Four cases died after birth and totally 39 fetuses remained until 12 months of age for ASQ assessment. 89.7% (35/39) showed normal development (group I), 5.1% (2/39) of the infants had minor neurologic deficiencies (group II), and 5.1% (2/39) had major neurologic deficiencies (group III). No difference was observed between recipient and donor status. A more favorable outcome was observed in single survivors (100% normal in the neurodevelopmental state) compared with double survivor twins (28/32, 87.5%) infants in group I (2/32, 6.25%) infants in group II, and (2/32, 6.25%) in group III. However, the difference was not significant (*p* = 0.243).

**Fig. 2:**
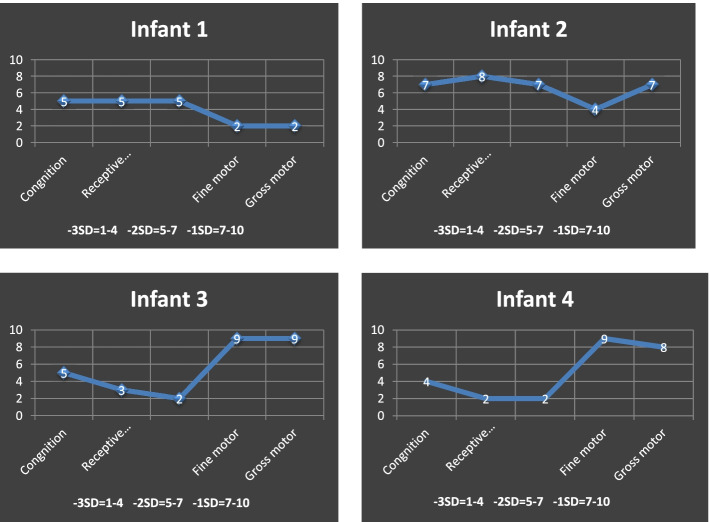
The justified scores of infants with neurodevelopmental impairment

Four infants were diagnosed with neurologic disorders, two of which had mild to moderate cerebral palsy (CP) and two had delayed verbal skills and autistic spectrum disorder. The infants’ characteristics are listed in Table 3. Twins with CP had severe combined immune deficiency (SCID), and as a result of recurrent respiratory infections and multiple hospitalizations, they had growth impairment and failure to thrive (FTT) and were waiting for bone marrow transplantation. Bayley examination results of the neonates are also listed in Table 4 and Fig. 2.

**Table 3:** Information of four infants with neurodevelopmental impairment

Case	Sex	Birth weight (gram)	Apgar 5^th min^	Stage	TTTS Classification	Gestational age at delivery (week)	Gestational age at laser (week)	Neurologic disorders	Comorbidity
1	Male	1800	7	III	Donor	34	23	Moderate spastic Diplegia CP	SCID, SGA, preterm birth
2	Male	2090	8	III	Recipient	34	23	Mild spastic Diplegia CP	SCID, preterm birth
3	Male	1850	8	II	Recipient	35	21 6/7	Autism spectrum	SGA, preterm birth
4	Male	1600	8	II	Donor	35	21 6/7	Autism spectrum	SGA, preterm birth

*SCID* Severe combined immune deficiency

**Table 4:** Bayley examination results in the neonates

Infants with NDI	Cognition Score	Motor (Composite Score)	Language (Composite Score)
Infant 1	**75**	**52**	**71**
Infant 2	**85**	**73**	**86**
Infant 3	**75**	**94**	**56**
Infant 4	**70**	**91**	**53**

The scores are within 0–100. The scores lower than 85 indicate mild impairment, and lower than 70 indicate moderate or severe impairment

## Discussion

In our study near to 90% of the cases showed normal development and 5% had major neurologic deficiencies of which 2 had mild to moderate cerebral palsy and two had delayed verbal skills and autistic spectrum disorder.

Advancing techniques including FLP in the management of TTTS twins necessitate the studies on their long-term neurodevelopment. Determining NDI includes a neurologic and physical examination and assessment of cognitive and motor development using developmental tests such as the Bayley Scales [5].

In a survey of 78 pregnant mothers who had undergone laser treatment 18% of the cases developed neurologic impairment [11]. In a similar study on 332 twins, no difference was noted between the incidence of neurologic impairment in the normal population and twins with TTTS [12]. Otherwise, NDI was not different according to the TTTS stages. Autism was found in two infants in our study (5%) which was the first report of this condition after FLC, although it is greater than general population (0.1%) [13]. It may be explained by the twinning process, which is a main risk factor for the development of autism [14]. The current study is the first that report autism after FLP in TTTS. Another study just reported the neurologic impairment in MCDA twins at school-age children (18). Also, in a survey of 177 infants showed that near to 10% had severe neurologic impairment [15] but autism was not reported in these studies. Considering the earlier birth of all the studied cases, it is not possible to announce FLC as a definite factor in the incidence of neurologic impairment in infants.

This is the first study from Iran that reported the neonatal outcomes and evaluated the neurodevelopmental outcomes of the infants with the history of TTTS undergoing fetoscopic laser photocoagulation and a longer follow up is another strength of this study. This is an ongoing study and gradually more infants are added for neurodevelopmental follow up. One of the main limitations of the current study is lack of adjustment for prematurity and birthweight which are considered as important variables affecting neurodevelopmental outcome.

## Conclusion

In our population, minor and major neurodevelopmental impairment were seen in 10.2% of the twin infants after FLC for twin-to-twin transfusion syndrome. This information is useful for counseling our couples in this population prior the procedure.

## Data Availability

The datasets used and/or analyzed during the current study are available from the corresponding author on reasonable request.
